# Supplementary material for: Perception and Attitude toward Teleconsultations among Different Healthcare Professionals in the Era of the COVID-19 Pandemic
Source: Int J Environ Res Public Health. 2022 Sep 13;19(18):11532. doi: 10.3390/ijerph191811532 (PMC9517420; doi:10.3390/ijerph191811532)
Supplement: Supplementary file 1 [file ijerph-19-11532-s001.zip › Supplementary File S1 ¿C A translated version of the questionnaire used in the study.pdf]

TELECONSULTATIONS IN HEALTHCARE

Please, complete the questionnaire regarding your opinion about the use of teleconsultations in health care setting. You can choose more than one answer in some of the questions.

METRICS OF THE RESPONDENT

Age:

◯ 20-30 years ◯ 31-40 years ◯ 41-50 years ◯ 51-60 years ◯ over 60 years

Sex:

◯ female ◯ male

Medical profession:

◯ doctor ◯ nurse ◯ physiotherapist ◯ paramedic ◯ midwife

Please enter your current specialization below:

....

PREFERENCES AND WORKING METHODS DURING COVID-19 PANDEMIC

Which method of work do you prefer during COVID-19 pandemic?

◯ teleconsultation ◯ personal visit

Did you use teleconsultations before the COVID-19 pandemic?

◯ yes ◯ no

How do you confirm the patient's identity during teleconsultation?

◯ I am asking for name and surname ◯ I am asking for home address

◯ I am asking for a personal identification number (PESEL) ◯ I am asking for a date of birth

◯ other methods: ....

What are the advantages of teleconsultations in your opinion?

◯ the reduced risk of contracting the SARS-CoV-2 virus for healthcare professionals

◯ the reduced risk of contracting the SARS-CoV-2 virus for the patients

◯ possibility of providing medical advice to more patients at the same time

◯ quick contact with patients

◯ other: ....

What are the disadvantages of teleconsultations in your opinion?

- ◯ impossibility to personally examine the patient
- ◯ unreliable transmission of information by the patient
- ◯ technical difficulties
- ◯ difficulties resulting from the patient's symptoms (e.g., deterioration of hearing, psychotic disorders)
- ◯ other ....

Do you have access to the results of laboratory tests, imaging tests, etc. during the teleconsultation?

◯ always ◯ often ◯ occasionally ◯ rarely ◯ never

How long does it take you to conduct a teleconsultation?

◯ the same as a personal visit ◯ less than a personal visit ◯ more than a personal visit

How do you assess in scale (from 1 to 10, where 1 is the lowest and 10 is the highest) effectiveness of teleconsultation?

	1	2	3	4	5	6	7	8	9	10	
the lowest effectiveness	◯	◯	◯	◯	◯	◯	◯	◯	◯	◯	the highest effectiveness

How do you assess in scale (from 1 to 10, where 1 is the lowest and 10 is the highest) reliability of teleconsultation?

	1	2	3	4	5	6	7	8	9	10	
the lowest reliability	◯	◯	◯	◯	◯	◯	◯	◯	◯	◯	the highest reliability

After the end of the COVID-19 pandemic, do you intend to use teleconsultation in your professional work?

◯ yes, often ◯ yes, occasionally ◯ no ◯ I have no opinion

What in your opinion, should be the ratio of the optimal number of teleconsultations to the personal visits in the primary care facilities?

- ◯ 0% teleconsultations – 100% personal visits
- ◯ 10% teleconsultations – 90% personal visits
- ◯ 20% teleconsultations – 80% personal visits
- ◯ 30% teleconsultations – 70% personal visits

◯ 40% teleconsultations – 60% personal visits

◯ 50% teleconsultations – 50% personal visits

◯ 60% teleconsultations – 40% personal visits

◯ 70% teleconsultations – 30% personal visits

◯ 80% teleconsultations – 20% personal visits

◯ 90% teleconsultations – 10% personal visits

◯ 100% teleconsultations – 0% personal visits

Own opinions, suggestions or observations about teleconsultations:

....

....
