# Supplementary material for: Perception and Attitude toward Teleconsultations among Different Healthcare Professionals in the Era of the COVID-19 Pandemic
Source: Int J Environ Res Public Health. 2022 Sep 13;19(18):11532. doi: 10.3390/ijerph191811532 (PMC9517420; doi:10.3390/ijerph191811532)
Supplement: Supplementary file 1 [file ijerph-19-11532-s001.zip › Supplementary File S2 ¿C Additional tables.pdf]

Table S1. The use of teleconsultation by Polish HCPs before, during and after the COVID-19 pandemic

Variable	Categories	n	%	χ2<br>Cramer's V	df	p
The preferred way of working during COVID-19 pandemic	Teleconsultation	388	50.5	0.06	1	0.801
	Personal visit	381	49.5	0.009		
Did you work before the COVID-19 pandemic using teleconsultation?	Yes	240	31.0	111.67	1	0
	No	534	69.0	0.380		
Do you have access to the results of laboratory tests, imaging tests, etc. during the teleconsultation?	Always	146	19.5	463.89	4	0
	Often	374	49.9	0.394		
	Occasionally	119	15.9			
	Rarely	43	5.7			
	Never	67	8.9			
How long does it take you to conduct a teleconsultation?	The same as a personal visit	247	33.4	93.7	2	0
	Less than a personal visit	354	47.8	0.252		
	More than a personal visit	139	18.8			
After the end of the COVID-19 pandemic, do you intend to use teleconsultation in your professional work?	Yes, often	202	26.5	403.97	3	0
	Yes, occasionally	407	53.4	0.420		
	No	121	15.9			
	I have no opinion	32	4.2			

Note. χ2 – the homogeneity test;, Cramers' V- a coefficient determining the level of dependence between two nominal variables, df – number of degrees of freedom, p - calculated significance level (value 0 means p <0.001)

Table S2. Availability to the test results during teleconsultation depending on the profession and specialization.

		**Availability to the test results during teleconsultation**										**Fisher's test Cramer's V**
		always		often		occasionally		rarely		never		
**Variable**	**Categories**	**n**	**%**	**n**	**%**	**n**	**%**	**n**	**%**	**n**	**%**	***p***
**Profession**	Doctor	123	22.7	315	58.2	72	13.3	14	2.6	17	3.1	**0**
	Nurse	17	12.7	41	30.6	27	20.1	15	11.2	34	25.4	0.245
	Physiotherapist	1	4.3	5	21.7	3	13.0	6	26.1	8	34.8	
	Paramedic	0	0.0	6	25.0	9	37.5	5	20.8	4	16.7	
	Midwife	5	18.5	7	25.9	8	29.6	3	11.1	4	14.8	
**Medical specialization***	Family medicine	68	26.2	168	64.6	19	7.3	1	0.4	4	1.5	**0**<br>0.244
	Internal diseases	7	13.7	22	43.1	10	19.6	3	5.9	9	17.6	
	Pediatrics	5	18.5	15	55.6	5	18.5	2	7.4	0	0.0	
	Cardiology	3	14.3	7	33.3	5	23.8	3	14.3	3	14.3	
	Hematology	2	20.0	7	70.0	1	10.0	0	0.0	0	0.0	
	Anesthesiology	1	9.1	2	18.2	3	27.3	0	0.0	5	45.5	
	Other	14	19.4	32	44.4	14	19.4	5	6.9	7	9.7	

Note. p - calculated significance level in Fisher's exact test of independence, p = 0 means that p <0.001; Cramers' V- a coefficient determining the level of dependence between two nominal variables ; * - the group of respondents was limited to those who indicated only one specialization

Table S3. Amount of time used for teleconsultation and personal visit in different age, gender and occupational groups among Polish HCPs.

		**Time used for teleconsultation in relation to a personal visit**						**Fisher's test Cramer's V**
		The same		Less		More		
**Variable**	**Categories**	**n**	**%**	**n**	**%**	**n**	**%**	***p***
Age	20-30 years	36	30.5	72	61.0	10	8.5	0.005
	31-40 years	76	32.9	116	50.2	39	16.9	0.117
	41-50 years	64	36.0	77	43.3	37	20.8	
	51-60 years	51	31.9	67	41.9	42	26.2	
	>60 years	20	37.7	22	41.5	11	20.8	
Sex	Female	194	35.5	258	47.2	95	17.4	0.071
	Male	53	27.5	96	49.7	44	22.8	0.084
Profession	Doctor	207	38.3	239	44.3	94	17.4	0
	Nurse	27	21.3	75	59.1	25	19.7	0.149
	Physiotherapist	2	8.7	11	47.8	10	43.5	
	Paramedic	4	16.7	17	70.8	3	12.5	
	Midwife	7	26.9	12	46.2	7	26.9	
Medical specialization*	Family medicine	105	40.4	122	46.9	33	12.7	0.337
	Internal diseases	20	40.8	21	42.9	8	16.3	0.124
	Pediatrics	11	40.7	11	40.7	5	18.5	
	Cardiology	4	19.0	15	71.4	2	9.5	
	Hematology	3	30.0	6	60.0	1	10.0	
	Anesthesiology	4	36.4	7	63.6	0	0.0	
	Other	20	27.8	37	51.4	15	20.8	

Note. p - calculated significance level in Fisher's exact test of independence, Cramers' V- a coefficient determining the level of dependence between two nominal variables; p = 0 means that p <0.001; * - the group of respondents was limited to those who indicated only one specialization

Table S4. The mean, standard deviation, median, values of the first and third quartiles and the confidence interval index for the effectiveness and reliability of the provided tele-advice according to the respondents.

**Variable**	**n**	**M**	**SD**	**Q.25%**	**Median**	**Q.75%**	**95% CI**
Effectiveness of conducted teleconsultation	765	6.19	2.29	5	7	8	6.49-6.99
Reliability of conducted teleconsultation	766	5.89	2.26	4	6	8	5.99-6.50

Note. Ratings on a scale of 1-10 (1 - lowest, 10 - highest). Q.25% - first quartile, Q.75% - third quartile; 95% CI - 95% confidence interval for the (pseudo) median.

Table S5. Assessment of the necessary ratio of teleconsultations to personal visits depending on the medical profession and medical specialization of the respondents

		**Assessment of the necessary ratio of teleconsultations to personal visits**																						**Fisher's test Cramer's V**
		0T-100P		10T-90P		20T-80P		30T-70P		40T-60P		50T-50P		60T-40P		70T-30P		80T-20P		90T-10P		100T-0P		
**Variable**	**Category**	n	%	n	%	n	%	n	%	n	%	n	%	n	%	n	%	n	%	n	%	n	%	*p*
**Profession**	Doctor	0	0.0	19	17.8	27	25.2	20	18.7	12	11.2	17	15.9	8	7.5	2	1.9	2	1.9	0	0	0	0	**0.005**
	Nurse	1	3.7	6	22.2	6	22.2	9	33.3	0	0.0	0	0.0	5	18.5	0	0.0	0	0.0	0	0	0	0	**-**
	Physiotherapist	1	11.1	4	44.4	4	44.4	0	0.0	0	0.0	0	0.0	0	0.0	0	0.0	0	0.0	0	0	0	0	
	Paramedic	0	0.0	3	100.0	0	0.0	0	0.0	0	0.0	0	0.0	0	0.0	0	0.0	0	0.0	0	0	0	0	
	Midwife	0	0.0	0	0.0	0	0.0	0	0.0	0	0.0	0	0.0	0	0.0	0	0.0	0	0.0	0	0	0	0	
**Medical specialization***	Family medicine	0	0.0	10	15.6	16	25.0	9	14.1	10	15.6	12	18.8	6	9.4	0	0.0	1	1.6	0	0	0	0	0.051<br>0.347
	Internal diseases	0	0.0	1	20.0	2	40.0	2	40.0	0	0.0	0	0.0	0	0.0	0	0.0	0	0.0	0	0	0	0	
	Pediatrics	0	0.0	2	66.7	0	0.0	0	0.0	1	33.3	0	0.0	0	0.0	0	0.0	0	0.0	0	0	0	0	
	Cardiology	0	0.0	0	0.0	1	50.0	1	50.0	0	0.0	0	0.0	0	0.0	0	0.0	0	0.0	0	0	0	0	
	Hematology	0	0.0	3	50.0	2	33.3	0	0.0	0	0.0	0	0.0	0	0.0	1	16.7	0	0.0	0	0	0	0	
	Anesthesiology	1	33.3	0	0.0	0	0.0	2	66.7	0	0.0	0	0.0	0	0.0	0	0.0	0	0.0	0	0	0	0	
	Other	1	10.0	4	40.0	1	10.0	3	30.0	0	0.0	0	0.0	1	10.0	0	0.0	0	0.0	0	0	0	0	

Note. p - calculated significance level in Fisher's test, p = 0 means that p <0.001; Cramers' V- a coefficient determining the level of dependence between two nominal variables; * - the group of respondents was limited to those who indicated only one specialization; T – percentage of teleconsultations, P – percentage of personal visits
